# The effect of voluntary fasting and dehydration on flicker-induced retinal vascular dilation in a healthy individual: a case report

**DOI:** 10.1186/1752-1947-2-153

**Published:** 2008-05-13

**Authors:** Rebekka Heitmar, Doina Gherghel, Richard Armstrong, Robert Cubbidge, Sarah Hosking

**Affiliations:** 1 School of Life and Health Sciences, Aston University, Birmingham, UK

## Abstract

**Introduction:**

Dynamic retinal vessel analysis represents a well-established method for the assessment of vascular reactivity during both normal conditions and after various provocations. We present a case where the subject showed abnormal retinal vessel reactivity after fasting voluntarily for 20 hours.

**Case presentation:**

A healthy, 21-year-old man who fasted voluntarily for 20 hours exhibited abnormal retinal vascular reactivity (dilation and constriction) after flicker provocation as measured using the Dynamic Retinal Vessel Analyser (Imedos, Jena, Germany).

**Conclusion:**

The abnormal vascular reactivity induced by fasting was significant; abnormal levels of important nutrients due to fasting and dehydration could play a role through altering the concentration of vasoactive substances such as nitric oxide. This hypothesis needs further investigation.

## Introduction

The assessment of retinal vessel diameters during both normal conditions and after various provocations could represent an important tool in investigating ocular diseases as well as for the initial diagnosis and subsequent followup of systemic disorders such as hypertension, cardiovascular disease and diabetes [1]. Since retinal vessel size is a major determinant of vascular resistance and hence of blood flow, any alterations in the diameter of this vascular bed could signal perfusion-related pathology occurring either locally or even systemically. Indeed, abnormalities of retinal vascular dynamics have been found in both ocular [2,3] and systemic vascular diseases [1,4].

## Case presentation

A 21-year-old healthy man presented voluntarily for a routine research appointment involving the assessment of retinal vessel reactivity using the Dynamic Retinal Vessel Analyser (DVA) at the Aston Academy of Life Sciences, Aston University, Birmingham. This device consists of a digital fundus camera combined with a CCD camera for electronic online image acquisition coupled to a video recorder for archiving [5]. During a routine experiment, a chosen vessel segment of approximately 500 μm is scanned at a rate of 25 Hz. After baseline measurements, flicker light is generated optoelectronically by chopping the fundus illumination at a frequency of 12.5 Hz.

After 20 minutes of room acclimatization to obtain stabile haemodynamic conditions, systemic blood pressure (BP) was measured three times (1 minute apart) using a manual sphygmomanometer. Systolic BP (SBP) and diastolic BP (DBP) values were obtained; the average readings for SBP and DBP were then used to calculate the mean arterial BP (MABP) using the formula: MABP = 2/3 × DBP+1/3 × SBP. After instilling one drop of oxybuprocaine hydrochloride 0.4% in the right eye, intraocular pressure (IOP) was also measured by means of a handheld contact tonometer. The IOP and MBP measurements were used to calculate the mean ocular perfusion pressure (MOPP) according to the formula: MOPP = 2/3 × MABP-IOP.

After full pupil dilation was reached by using tropicamide 1.0% retinal diameters of the superior temporal retinal artery and vein, measured approximately 1.5 disc diameters away from the optic nerve head, were assessed continuously over 350 seconds by using the DVA machine according to a previously established protocol [6]. Briefly, the measurement steps were: 50 seconds of still illumination (baseline recording) followed by three cycles of 20 seconds of flicker stimulation interspersed with 80 seconds still illumination (representing recovery time). The main measured outcome is the vessel width expressed in units of measurement; for the stimulation with flicker light, the outcome was defined as percent changed to baseline. In addition to this standard measure, the time needed to reach maximum dilation in both the artery and the vein during fasting and normal conditions (called the 'reaction time') was calculated.

**Figure 1:**
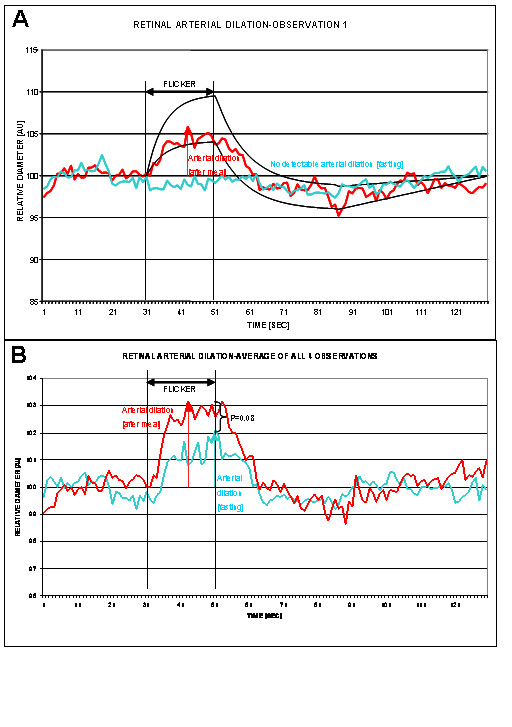
**(A) **Initial measurement of retinal arteriole diameter under fasting and normal conditions (blue and red, respectively), (black lines showing normal range in healthy subjects). **(B) **Average curve of all four observations under fasting and normal conditions (blue and red, respectively).

**Figure 2:**
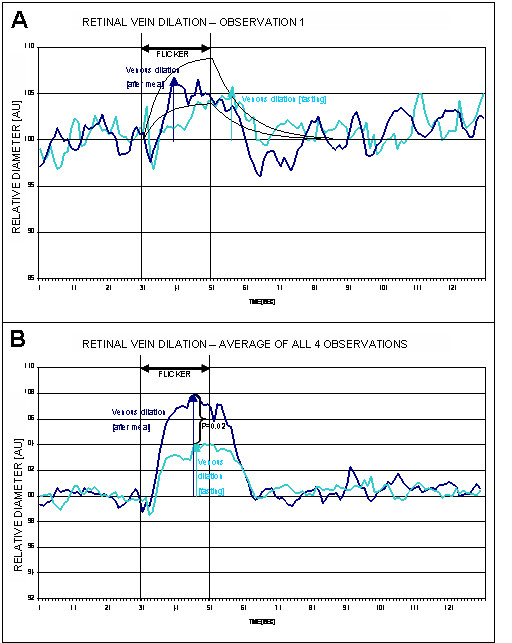
**(A) **Initial measurement of retinal venule diameter under fasting and normal conditions (light blue and dark blue, respectively), (black lines showing upper and lower confidence limits in healthy subjects). **(B) **Average curve of all four observations under fasting and normal conditions (light blue and dark blue, respectively).

The results of the initial measurements under fasting conditions and after a meal are shown in Table 1, and Figure 1A and Figure 2A. As compared with what is expected in a healthy subject under normal conditions [1,6] our results have shown a subnormal retinal vessels response in both the arteriole (mean dilation = 2.59 ± 1.98%) and venule (mean dilation = 5.19 ± 2.53%). This observation has triggered a more detailed history of the patient's background and dietary habits; this later step revealed that the subject had been voluntarily fasting for 20 hours prior to the research appointment. In order to enable us to see if this particular aspect has had any influence on the measurements, the subject was sent to have a meal and all measurements were repeated immediately afterwards.

**Table 1:** Showing retinal venule and arteriole dilation after each cycle of flickering light stimulation, as change in percentage, compared to baseline diameter as measured under fasting conditions.

CONDITION: FASTING	
FLICKER STIMULATION	VENOUS DILATION (in % to baseline value)

F1	3.90
F2	8.29
F3	4.47
MEAN +/- STD	5.59 +/- 2.37

	ARTERIAL DILATION (in % to baseline value)

F1	5.20
F2	3.96
F3	4.72
MEAN +/- STD	4.63 +/- 0.63

The results of the second set of measurements are shown in Table 2. The difference between the measurements performed before and after the meal were statistically significant for the retinal venous dilation (p = 0.03) but not for the retinal artery dilation (p = 0.08).

**Table 2:** Showing retinal venule and arteriole dilation after each cycle of flickering light stimulation, as change in percentage, compared to baseline diameter as measured immediately after a meal.

CONDITION: AFTER MEAL	
FLICKER STIMULATION	VENOUS DILATION (in % to baseline value)

F1	11.91
F2	5.26
F3	8.16
MEAN +/- STD	8.45 +/- 3.34

	ARTERIAL DILATION (in % to baseline value)

F1	9.18
F2	8.76
F3	11.69
MEAN +/- STD	9.88 +/- 1.58

**Figure 3:**
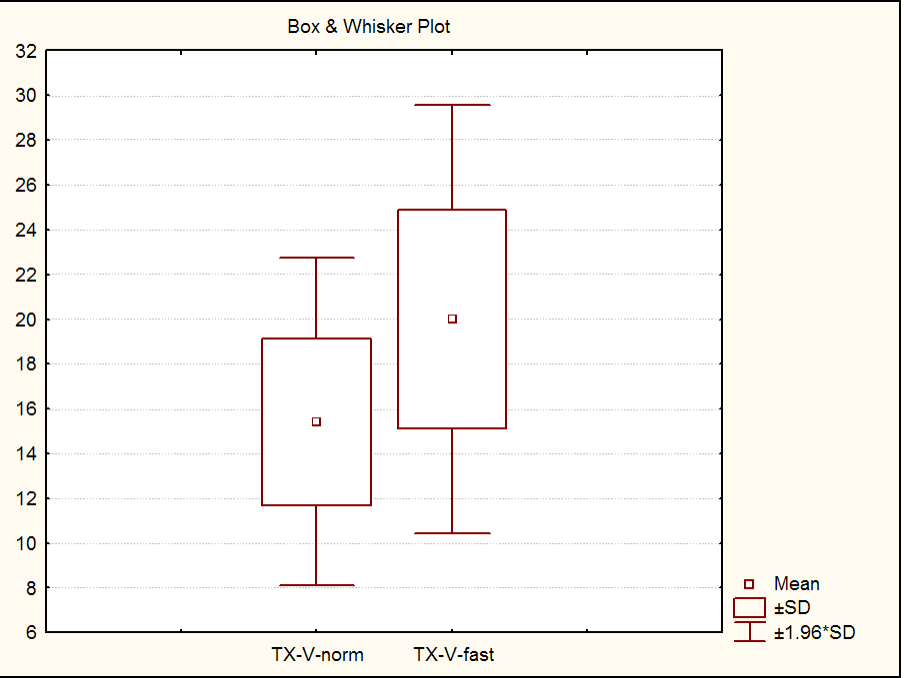
Retinal venule reaction time under normal conditions (TX-V-norm) and under fasting conditions (TX-V-fast).

With the subject's consent, the experiment was repeated on three additional occasions, each one month apart. During these subsequent tests, the subject was in a similar physiological condition to the initial experiment. Results of all observations are shown in Table 3, and Figures 1B, 2B and 3. Retinal vein dilation and reaction time, but not artery dilation and reaction time, were statistically significantly lower in fasting versus non-fasting conditions (dilation: p = 0.02 and p = 0.08; reaction time: p = 0.03 and p > 0.05, respectively). These results were comparable with the first observation that triggered the subsequent tests (a blunted vascular response during fasting and a normal response during after a meal).

**Table 3:** Showing results of all 4 observations along with p-values, as obtained from the statistical analysis.

	CONDITION: FASTING	CONDITION: AFTER MEAL	P-VALUE
VENOUS DILATION [% change to baseline]	5.19 +/- 2.53	9.01 +/- 2.69	**0.02**
ARTERIAL DILATION [% change to baseline]	2.59 +/- 1.98	3.91 +/- 2.41	0.08
REACTION TIME [sec]	20 +/- 4.88	15.41 +/- 3.72	**0.03**

Although SBP was statistically different when measured in the fasting versus non-fasting state (111.5 ± 1.17; 113.5 ± 1.17; p = 0.01) the MABP, IOP and OPP were not statistically different in the fasting versus non-fasting state (p = 0.24, p = 0.11 and p = 0.64, respectively).

## Discussion

This case demonstrates for the first time that despite having comparable intraocular and systemic 'pressure' conditions, the retinal vascular reactivity was blunted during voluntary fasting in a healthy, young individual. This observation was repeatable on four separate occasions.

The ocular circulation has a very complex regulating system able to maintain constant blood flow and oxygen supply to the tissue despite variations in systemic BP [1,7]. Autonomic nervous systems, as well as endothelial, metabolic, myogenic and neurogenic factors play important roles at different levels in this complex process. Molecules such as nitric oxide (NO), arginine and glucose also intervene by regulating smooth muscle cell relaxation, and hence vascular dilation [1,8,9].

The vascular response to flickering light has been widely studied in both animals and humans [1,6]. This provocation results in increased metabolic demand and, therefore, activation of NO synthase with subsequent vasodilation occurs [8,10]. It is therefore possible that the abnormal vascular dilation reported here, may be the result of low NO availability; however, the mechanism resulting in NO depletion can only be hypothesized at this stage. Changes in the levels of active substances such as lactate and glucose could also play role [8,10-12] in the vascular response to flicker provocation. Therefore, beside modifications in the NO dynamics, practically any change in the quantity and composition of the circulating fluid due to dehydration and/or fasting is also capable of altering vascular dynamics and BP [13]. Indeed, Alghadyan et al. [14] found a significant increase in onset of retinal vein occlusion (RVO) during fasting, suggesting a possible relationship between fasting and dehydration in the pathogenesis of RVO. Moreover, Lapeyraque et al. reported about five cases of chronic hypovolaemia occurring during continuous peritoneal dialysis which could result in blindness due to ocular ischaemia [15].

Although the data obtained in this case study is limited and needs further investigation to demonstrate the exact mechanism behind our observation, it clearly shows the clinical significance of detailed medical history before conducting and interpreting any test results. Knowledge of medical and/or family history and ethnic background at the time of examination is of great importance. In addition, since fluid and/or nutrient intake plays an important role in the physiology of important variables such as BP, vessel dynamics and blood composition, which are commonly used in diagnosing various systemic vascular disorders, fasting and dehydration could act as confounding factors and the clinical and/or laboratory results could be misleading.

## Conclusion

We present a case of decreased retinal vascular reactivity due to fasting and dehydration in a young and healthy man. This scenario could represent a potential case of vasoactive substance imbalance resulting in vasodilatory inhibition at the level of retinal vessels. The importance of this finding should be investigated in further studies and additional tests should be included to verify our hypothesis.

## Abbreviations

BP: blood pressure; DBP: diastolic blood pressure; DVA: dynamic retinal vessel analyser; IOP: intraocular pressure; MABP: mean arterial blood pressure; MOPP: mean ocular perfusion pressure; NO: nitric oxide; RVO: retinal vein occlusion; SBP: systolic blood pressure.

## Competing interests

The authors declare that they have no competing interests.

## Authors' contributions

RH collected the data, was involved in data analysis and interpretation and drafting of the manuscript. DG revised the manuscript and was involved in the data analysis and interpretation. RA was involved in the statistical analysis and interpretation. RC made substantial contributions to the design and collection. SH made substantial contributions to the design and collection. All authors read and approved the final manuscript.

## Consent

Written informed consent was obtained from the patient for publication of this case report and any accompanying images. A copy of the written consent is available for review by the Editor-in-Chief of this journal.
